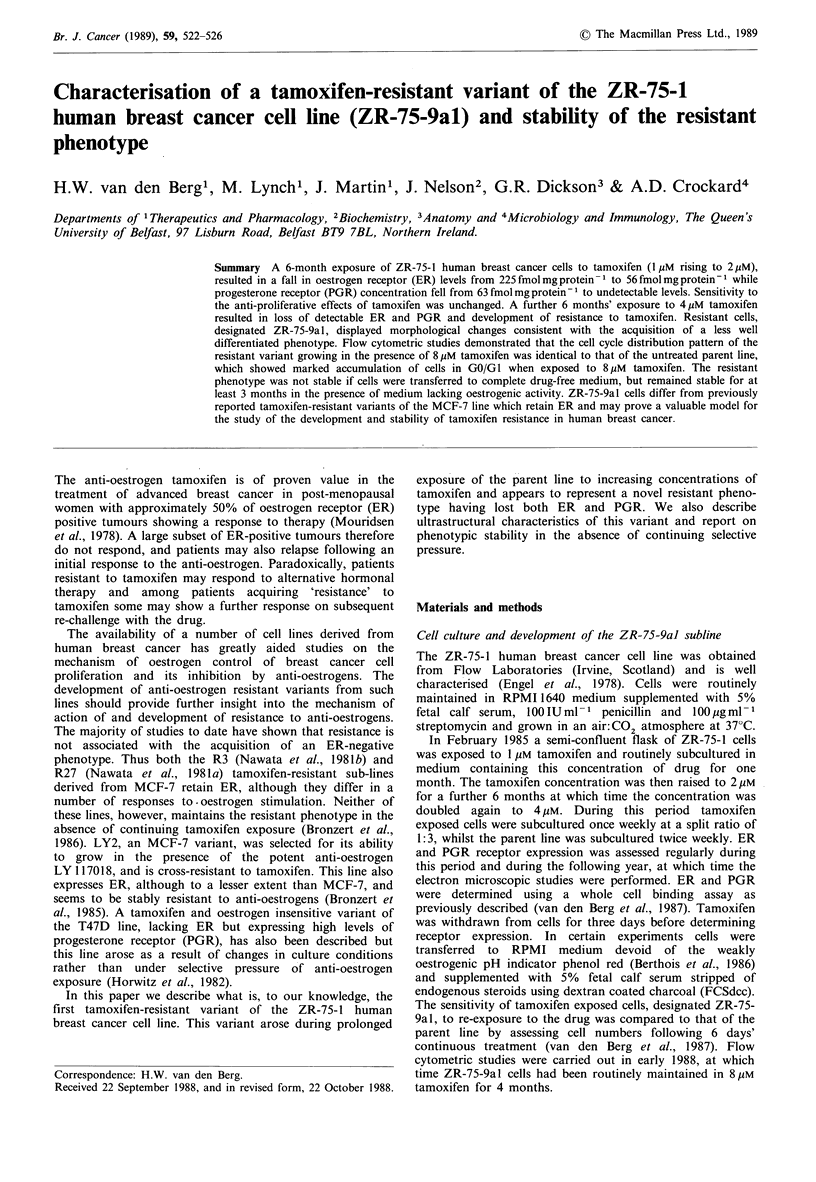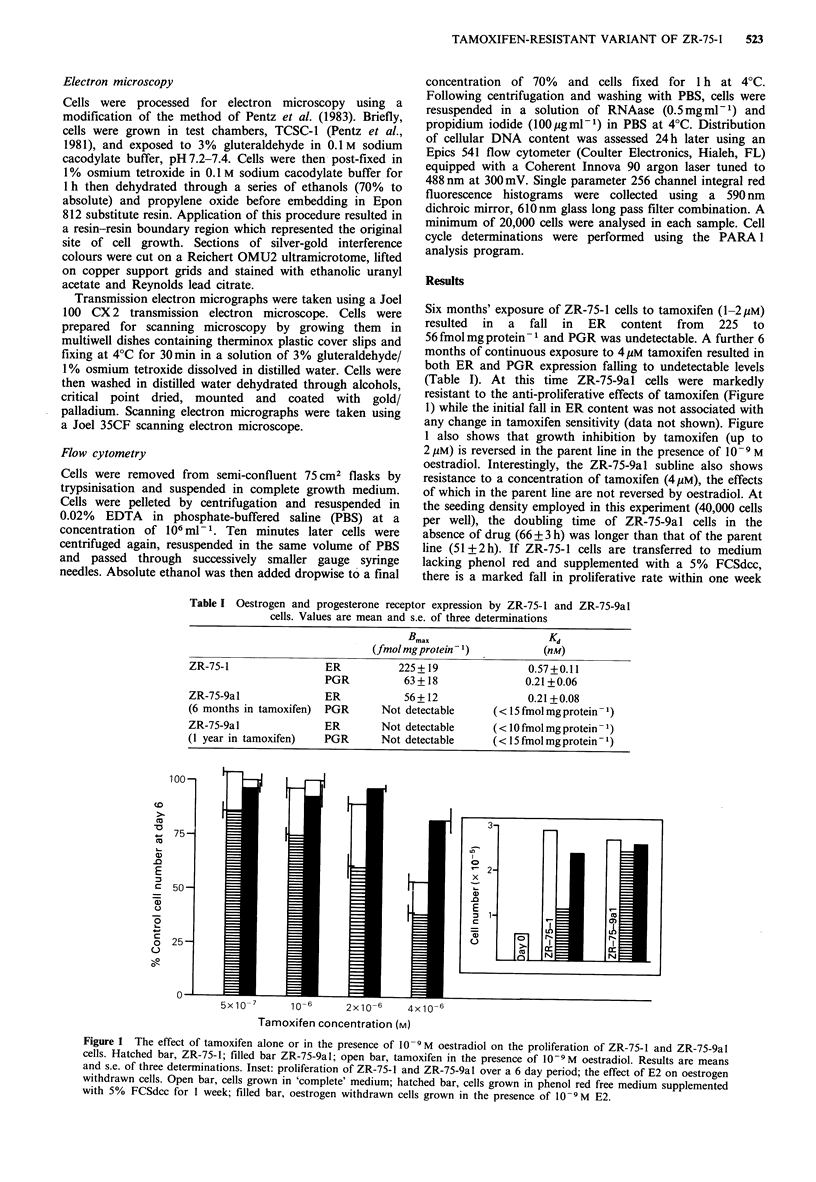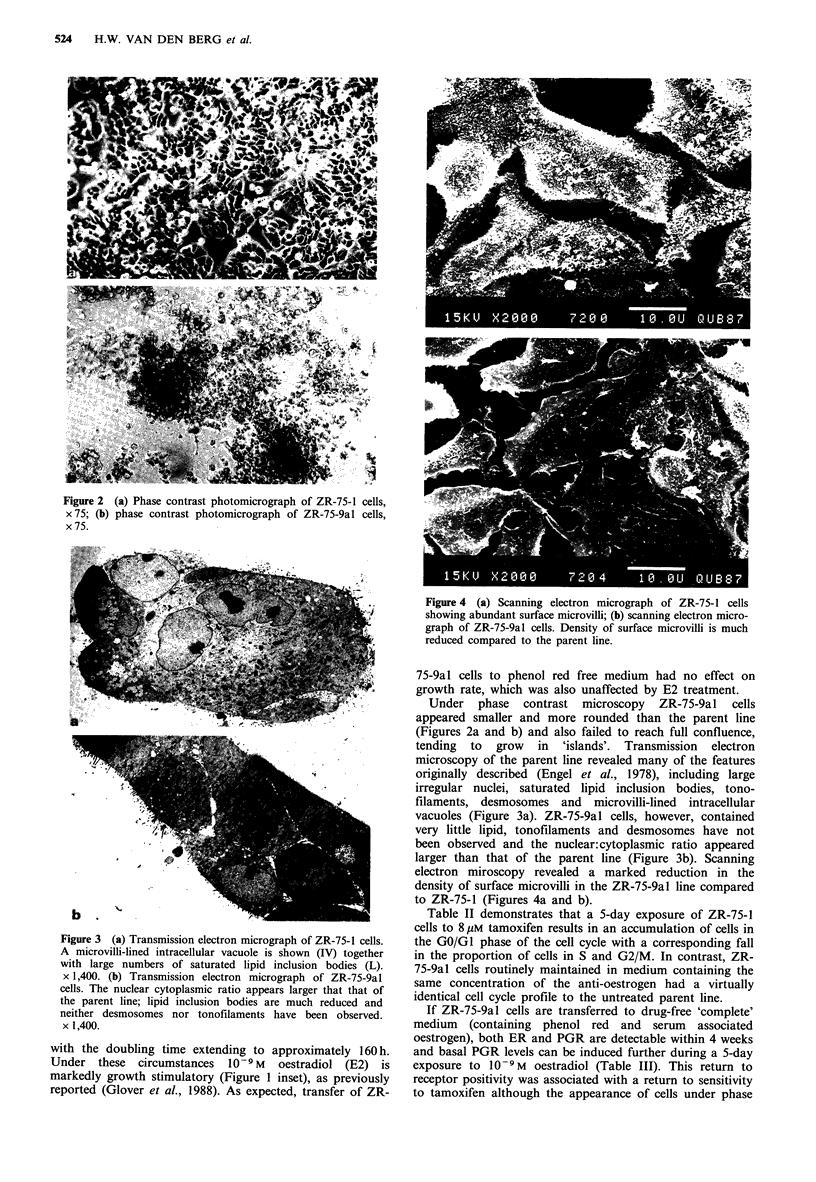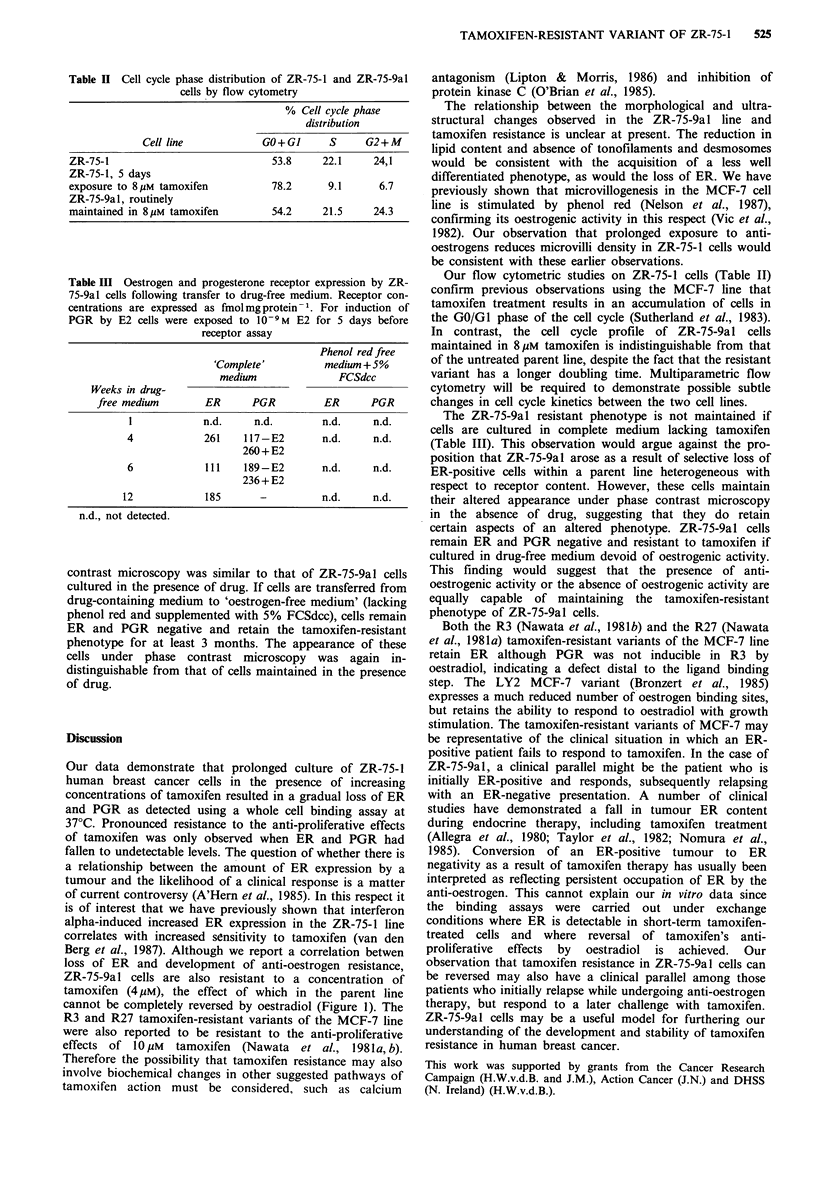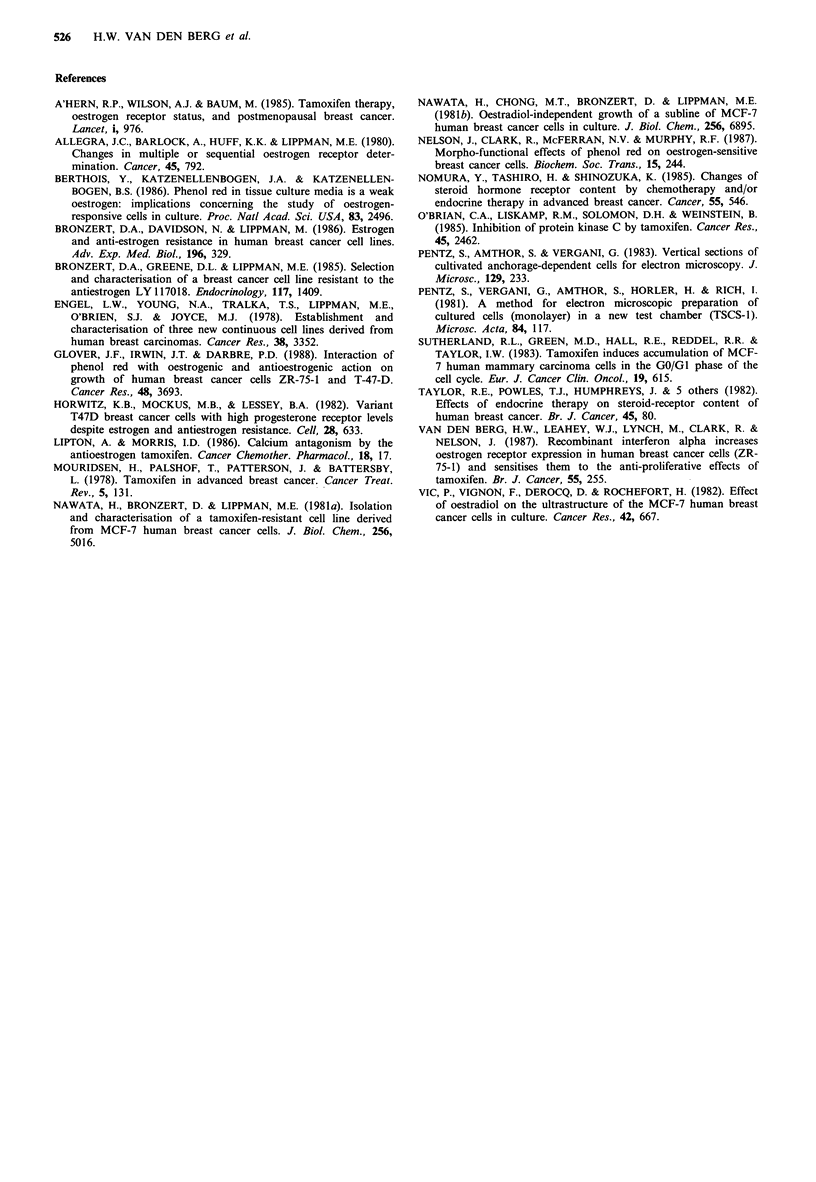# Characterisation of a tamoxifen-resistant variant of the ZR-75-1 human breast cancer cell line (ZR-75-9a1) and ability of the resistant phenotype.

**DOI:** 10.1038/bjc.1989.107

**Published:** 1989-04

**Authors:** H. W. van den Berg, M. Lynch, J. Martin, J. Nelson, G. R. Dickson, A. D. Crockard

**Affiliations:** Department of Therapeutics and Pharmacology, Queen's University of Belfast, Northern Ireland.

## Abstract

**Images:**


Br. J. Cancer (1989), 59, 522–526 © The Macmillan Press Ltd., 1989

### Characterisation of a tamoxifen-resistant variant of the ZR-75-1 human breast cancer cell line (ZR-75-9a1) and stability of the resistant phenotype

H.W. van den Berg[1], M. Lynch[1], J. Martin[1], J. Nelson[2], G.R. Dickson[3] & A.D. Crockard[4]

*Departments of [1]Therapeutics and Pharmacology, [2]Biochemistry, [3]Anatomy and [4]Microbiology and Immunology, The Queen's University of Belfast, 97 Lisburn Road, Belfast BT9 7BL, Northern Ireland.*

**Summary** A 6-month exposure of ZR-75-1 human breast cancer cells to tamoxifen (1 $\mu$M rising to 2 $\mu$M), resulted in a fall in oestrogen receptor (ER) levels from 225 fmol mg protein$^{-1}$ to 56 fmol mg protein$^{-1}$ while progesterone receptor (PGR) concentration fell from 63 fmol mg protein$^{-1}$ to undetectable levels. Sensitivity to the anti-proliferative effects of tamoxifen was unchanged. A further 6 months' exposure to 4 $\mu$M tamoxifen resulted in loss of detectable ER and PGR and development of resistance to tamoxifen. Resistant cells, designated ZR-75-9a1, displayed morphological changes consistent with the acquisition of a less well differentiated phenotype. Flow cytometric studies demonstrated that the cell cycle distribution pattern of the resistant variant growing in the presence of 8 $\mu$M tamoxifen was identical to that of the untreated parent line, which showed marked accumulation of cells in G0/G1 when exposed to 8 $\mu$M tamoxifen. The resistant phenotype was not stable if cells were transferred to complete drug-free medium, but remained stable for at least 3 months in the presence of medium lacking oestrogenic activity. ZR-75-9a1 cells differ from previously reported tamoxifen-resistant variants of the MCF-7 line which retain ER and may prove a valuable model for the study of the development and stability of tamoxifen resistance in human breast cancer.

The anti-oestrogen tamoxifen is of proven value in the treatment of advanced breast cancer in post-menopausal women with approximately 50% of oestrogen receptor (ER) positive tumours showing a response to therapy (Mouridsen *et al.*, 1978). A large subset of ER-positive tumours therefore do not respond, and patients may also relapse following an initial response to the anti-oestrogen. Paradoxically, patients resistant to tamoxifen may respond to alternative hormonal therapy and among patients acquiring 'resistance' to tamoxifen some may show a further response on subsequent re-challenge with the drug.

The availability of a number of cell lines derived from human breast cancer has greatly aided studies on the mechanism of oestrogen control of breast cancer cell proliferation and its inhibition by anti-oestrogens. The development of anti-oestrogen resistant variants from such lines should provide further insight into the mechanism of action of and development of resistance to anti-oestrogens. The majority of studies to date have shown that resistance is not associated with the acquisition of an ER-negative phenotype. Thus both the R3 (Nawata *et al.*, 1981*b*) and R27 (Nawata *et al.*, 1981*a*) tamoxifen-resistant sub-lines derived from MCF-7 retain ER, although they differ in a number of responses to oestrogen stimulation. Neither of these lines, however, maintains the resistant phenotype in the absence of continuing tamoxifen exposure (Bronzert *et al.*, 1986). LY2, an MCF-7 variant, was selected for its ability to grow in the presence of the potent anti-oestrogen LY 117018, and is cross-resistant to tamoxifen. This line also expresses ER, although to a lesser extent than MCF-7, and seems to be stably resistant to anti-oestrogens (Bronzert *et al.*, 1985). A tamoxifen and oestrogen insensitive variant of the T47D line, lacking ER but expressing high levels of progesterone receptor (PGR), has also been described but this line arose as a result of changes in culture conditions rather than under selective pressure of anti-oestrogen exposure (Horwitz *et al.*, 1982).

In this paper we describe what is, to our knowledge, the first tamoxifen-resistant variant of the ZR-75-1 human breast cancer cell line. This variant arose during prolonged exposure of the parent line to increasing concentrations of tamoxifen and appears to represent a novel resistant phenotype having lost both ER and PGR. We also describe ultrastructural characteristics of this variant and report on phenotypic stability in the absence of continuing selective pressure.

Correspondence: H.W. van den Berg.
Received 22 September 1988, and in revised form, 22 October 1988.

### Materials and methods

### *Cell culture and development of the ZR-75-9a1 subline*

The ZR-75-1 human breast cancer cell line was obtained from Flow Laboratories (Irvine, Scotland) and is well characterised (Engel *et al.*, 1978). Cells were routinely maintained in RPMI 1640 medium supplemented with 5% fetal calf serum, 100 IU ml$^{-1}$ penicillin and 100 $\mu$g ml$^{-1}$ streptomycin and grown in an air:$CO_2$ atmosphere at 37°C.

In February 1985 a semi-confluent flask of ZR-75-1 cells was exposed to 1 $\mu$M tamoxifen and routinely subcultured in medium containing this concentration of drug for one month. The tamoxifen concentration was then raised to 2 $\mu$M for a further 6 months at which time the concentration was doubled again to 4 $\mu$M. During this period tamoxifen exposed cells were subcultured once weekly at a split ratio of 1:3, whilst the parent line was subcultured twice weekly. ER and PGR receptor expression was assessed regularly during this period and during the following year, at which time the electron microscopic studies were performed. ER and PGR were determined using a whole cell binding assay as previously described (van den Berg *et al.*, 1987). Tamoxifen was withdrawn from cells for three days before determining receptor expression. In certain experiments cells were transferred to RPMI medium devoid of the weakly oestrogenic pH indicator phenol red (Berthois *et al.*, 1986) and supplemented with 5% fetal calf serum stripped of endogenous steroids using dextran coated charcoal (FCSdcc). The sensitivity of tamoxifen exposed cells, designated ZR-75-9a1, to re-exposure to the drug was compared to that of the parent line by assessing cell numbers following 6 days' continuous treatment (van den Berg *et al.*, 1987). Flow cytometric studies were carried out in early 1988, at which time ZR-75-9a1 cells had been routinely maintained in 8 $\mu$M tamoxifen for 4 months.

*Electron microscopy*

Cells were processed for electron microscopy using a modification of the method of Pentz *et al.* (1983). Briefly, cells were grown in test chambers, TCSC-1 (Pentz *et al.*, 1981), and exposed to 3% gluteraldehyde in 0.1 M sodium cacodylate buffer, pH 7.2–7.4. Cells were then post-fixed in 1% osmium tetroxide in 0.1 M sodium cacodylate buffer for 1 h then dehydrated through a series of ethanols (70% to absolute) and propylene oxide before embedding in Epon 812 substitute resin. Application of this procedure resulted in a resin–resin boundary region which represented the original site of cell growth. Sections of silver-gold interference colours were cut on a Reichert OMU2 ultramicrotome, lifted on copper support grids and stained with ethanolic uranyl acetate and Reynolds lead citrate.

Transmission electron micrographs were taken using a Joel 100 CX2 transmission electron microscope. Cells were prepared for scanning microscopy by growing them in multiwell dishes containing therminox plastic cover slips and fixing at 4°C for 30 min in a solution of 3% gluteraldehyde/1% osmium tetroxide dissolved in distilled water. Cells were then washed in distilled water dehydrated through alcohols, critical point dried, mounted and coated with gold/palladium. Scanning electron micrographs were taken using a Joel 35CF scanning electron microscope.

*Flow cytometry*

Cells were removed from semi-confluent 75 $cm^2$ flasks by trypsinisation and suspended in complete growth medium. Cells were pelleted by centrifugation and resuspended in 0.02% EDTA in phosphate-buffered saline (PBS) at a concentration of $10^6 ml^{-1}$. Ten minutes later cells were centrifuged again, resuspended in the same volume of PBS and passed through successively smaller gauge syringe needles. Absolute ethanol was then added dropwise to a final concentration of 70% and cells fixed for 1 h at 4°C. Following centrifugation and washing with PBS, cells were resuspended in a solution of RNAase (0.5 $mg ml^{-1}$) and propidium iodide (100 $\mu g ml^{-1}$) in PBS at 4°C. Distribution of cellular DNA content was assessed 24 h later using an Epics 541 flow cytometer (Coulter Electronics, Hialeh, FL) equipped with a Coherent Innova 90 argon laser tuned to 488 nm at 300 mV. Single parameter 256 channel integral red fluorescence histograms were collected using a 590 nm dichroic mirror, 610 nm glass long pass filter combination. A minimum of 20,000 cells were analysed in each sample. Cell cycle determinations were performed using the PARA 1 analysis program.

### Results

Six months' exposure of ZR-75-1 cells to tamoxifen (1–2 $\mu$M) resulted in a fall in ER content from 225 to 56 fmol mg $protein^{-1}$ and PGR was undetectable. A further 6 months of continuous exposure to 4 $\mu$M tamoxifen resulted in both ER and PGR expression falling to undetectable levels (Table I). At this time ZR-75-9a1 cells were markedly resistant to the anti-proliferative effects of tamoxifen (Figure 1) while the initial fall in ER content was not associated with any change in tamoxifen sensitivity (data not shown). Figure 1 also shows that growth inhibition by tamoxifen (up to 2 $\mu$M) is reversed in the parent line in the presence of $10^{-9}$ M oestradiol. Interestingly, the ZR-75-9a1 subline also shows resistance to a concentration of tamoxifen (4 $\mu$M), the effects of which in the parent line are not reversed by oestradiol. At the seeding density employed in this experiment (40,000 cells per well), the doubling time of ZR-75-9a1 cells in the absence of drug (66 ± 3 h) was longer than that of the parent line (51 ± 2 h). If ZR-75-1 cells are transferred to medium lacking phenol red and supplemented with a 5% FCSdcc, there is a marked fall in proliferative rate within one week

**Table I** Oestrogen and progesterone receptor expression by ZR-75-1 and ZR-75-9a1 cells. Values are mean and s.e. of three determinations

| | | $B_{max}$ (*fmol mg protein*$^{-1}$) | $K_d$ (*nM*) |
|---|---|---|---|
| ZR-75-1 | ER | 225 ± 19 | 0.57 ± 0.11 |
| | PGR | 63 ± 18 | 0.21 ± 0.06 |
| ZR-75-9a1 | ER | 56 ± 12 | 0.21 ± 0.08 |
| (6 months in tamoxifen) | PGR | Not detectable | (< 15 fmol mg $protein^{-1}$) |
| ZR-75-9a1 | ER | Not detectable | (< 10 fmol mg $protein^{-1}$) |
| (1 year in tamoxifen) | PGR | Not detectable | (< 15 fmol mg $protein^{-1}$) |

**Figure 1** The effect of tamoxifen alone or in the presence of $10^{-9}$ M oestradiol on the proliferation of ZR-75-1 and ZR-75-9a1 cells. Hatched bar, ZR-75-1; filled bar ZR-75-9a1; open bar, tamoxifen in the presence of $10^{-9}$ M oestradiol. Results are means and s.e. of three determinations. Inset: proliferation of ZR-75-1 and ZR-75-9a1 over a 6 day period; the effect of E2 on oestrogen withdrawn cells. Open bar, cells grown in 'complete' medium; hatched bar, cells grown in phenol red free medium supplemented with 5% FCSdcc for 1 week; filled bar, oestrogen withdrawn cells grown in the presence of $10^{-9}$ M E2.

**Figure 2** (a) Phase contrast photomicrograph of ZR-75-1 cells, ×75; (b) phase contrast photomicrograph of ZR-75-9a1 cells, ×75.

**Figure 3** (a) Transmission electron micrograph of ZR-75-1 cells. A microvilli-lined intracellular vacuole is shown (IV) together with large numbers of saturated lipid inclusion bodies (L). ×1,400. (b) Transmission electron micrograph of ZR-75-9a1 cells. The nuclear cytoplasmic ratio appears larger that that of the parent line; lipid inclusion bodies are much reduced and neither desmosomes nor tonofilaments have been observed. ×1,400.

with the doubling time extending to approximately 160 h. Under these circumstances $10^{-9}$ M oestradiol (E2) is markedly growth stimulatory (Figure 1 inset), as previously reported (Glover et al., 1988). As expected, transfer of ZR-

**Figure 4** (a) Scanning electron micrograph of ZR-75-1 cells showing abundant surface microvilli; (b) scanning electron micrograph of ZR-75-9a1 cells. Density of surface microvilli is much reduced compared to the parent line.

75-9a1 cells to phenol red free medium had no effect on growth rate, which was also unaffected by E2 treatment.

Under phase contrast microscopy ZR-75-9a1 cells appeared smaller and more rounded than the parent line (Figures 2a and b) and also failed to reach full confluence, tending to grow in 'islands'. Transmission electron microscopy of the parent line revealed many of the features originally described (Engel et al., 1978), including large irregular nuclei, saturated lipid inclusion bodies, tonofilaments, desmosomes and microvilli-lined intracellular vacuoles (Figure 3a). ZR-75-9a1 cells, however, contained very little lipid, tonofilaments and desmosomes have not been observed and the nuclear:cytoplasmic ratio appeared larger than that of the parent line (Figure 3b). Scanning electron miroscopy revealed a marked reduction in the density of surface microvilli in the ZR-75-9a1 line compared to ZR-75-1 (Figures 4a and b).

Table II demonstrates that a 5-day exposure of ZR-75-1 cells to 8 $\mu$M tamoxifen results in an accumulation of cells in the G0/G1 phase of the cell cycle with a corresponding fall in the proportion of cells in S and G2/M. In contrast, ZR-75-9a1 cells routinely maintained in medium containing the same concentration of the anti-oestrogen had a virtually identical cell cycle profile to the untreated parent line.

If ZR-75-9a1 cells are transferred to drug-free 'complete' medium (containing phenol red and serum associated oestrogen), both ER and PGR are detectable within 4 weeks and basal PGR levels can be induced further during a 5-day exposure to $10^{-9}$ M oestradiol (Table III). This return to receptor positivity was associated with a return to sensitivity to tamoxifen although the appearance of cells under phase

**Table II** Cell cycle phase distribution of ZR-75-1 and ZR-75-9a1 cells by flow cytometry

| *Cell line* | *% Cell cycle phase distribution* | | |
|---|---|---|---|
| | *G0+G1* | *S* | *G2+M* |
| ZR-75-1 | 53.8 | 22.1 | 24,1 |
| ZR-75-1, 5 days exposure to 8 μM tamoxifen | 78.2 | 9.1 | 6.7 |
| ZR-75-9a1, routinely maintained in 8 μM tamoxifen | 54.2 | 21.5 | 24.3 |

**Table III** Oestrogen and progesterone receptor expression by ZR-75-9a1 cells following transfer to drug-free medium. Receptor concentrations are expressed as fmol mg protein$^{-1}$. For induction of PGR by E2 cells were exposed to $10^{-9}$ M E2 for 5 days before receptor assay

| *Weeks in drug-free medium* | *'Complete' medium* | | *Phenol red free medium+5% FCSdcc* | |
|---|---|---|---|---|
| | *ER* | *PGR* | *ER* | *PGR* |
| 1 | n.d. | n.d. | n.d. | n.d. |
| 4 | 261 | 117−E2<br>260+E2 | n.d. | n.d. |
| 6 | 111 | 189−E2<br>236+E2 | n.d. | n.d. |
| 12 | 185 | - | n.d. | n.d. |

n.d., not detected.

contrast microscopy was similar to that of ZR-75-9a1 cells cultured in the presence of drug. If cells are transferred from drug-containing medium to 'oestrogen-free medium' (lacking phenol red and supplemented with 5% FCSdcc), cells remain ER and PGR negative and retain the tamoxifen-resistant phenotype for at least 3 months. The appearance of these cells under phase contrast microscopy was again indistinguishable from that of cells maintained in the presence of drug.

### Discussion

Our data demonstrate that prolonged culture of ZR-75-1 human breast cancer cells in the presence of increasing concentrations of tamoxifen resulted in a gradual loss of ER and PGR as detected using a whole cell binding assay at 37°C. Pronounced resistance to the anti-proliferative effects of tamoxifen was only observed when ER and PGR had fallen to undetectable levels. The question of whether there is a relationship between the amount of ER expression by a tumour and the likelihood of a clinical response is a matter of current controversy (A'Hern *et al.*, 1985). In this respect it is of interest that we have previously shown that interferon alpha-induced increased ER expression in the ZR-75-1 line correlates with increased sensitivity to tamoxifen (van den Berg *et al.*, 1987). Although we report a correlation betwen loss of ER and development of anti-oestrogen resistance, ZR-75-9a1 cells are also resistant to a concentration of tamoxifen (4 μM), the effect of which in the parent line cannot be completely reversed by oestradiol (Figure 1). The R3 and R27 tamoxifen-resistant variants of the MCF-7 line were also reported to be resistant to the anti-proliferative effects of 10 μM tamoxifen (Nawata *et al.*, 1981*a*, *b*). Therefore the possibility that tamoxifen resistance may also involve biochemical changes in other suggested pathways of tamoxifen action must be considered, such as calcium antagonism (Lipton & Morris, 1986) and inhibition of protein kinase C (O'Brian *et al.*, 1985).

The relationship between the morphological and ultrastructural changes observed in the ZR-75-9a1 line and tamoxifen resistance is unclear at present. The reduction in lipid content and absence of tonofilaments and desmosomes would be consistent with the acquisition of a less well differentiated phenotype, as would the loss of ER. We have previously shown that microvillogenesis in the MCF-7 cell line is stimulated by phenol red (Nelson *et al.*, 1987), confirming its oestrogenic activity in this respect (Vic *et al.*, 1982). Our observation that prolonged exposure to anti-oestrogens reduces microvilli density in ZR-75-1 cells would be consistent with these earlier observations.

Our flow cytometric studies on ZR-75-1 cells (Table II) confirm previous observations using the MCF-7 line that tamoxifen treatment results in an accumulation of cells in the G0/G1 phase of the cell cycle (Sutherland *et al.*, 1983). In contrast, the cell cycle profile of ZR-75-9a1 cells maintained in 8 μM tamoxifen is indistinguishable from that of the untreated parent line, despite the fact that the resistant variant has a longer doubling time. Multiparametric flow cytometry will be required to demonstrate possible subtle changes in cell cycle kinetics between the two cell lines.

The ZR-75-9a1 resistant phenotype is not maintained if cells are cultured in complete medium lacking tamoxifen (Table III). This observation would argue against the proposition that ZR-75-9a1 arose as a result of selective loss of ER-positive cells within a parent line heterogeneous with respect to receptor content. However, these cells maintain their altered appearance under phase contrast microscopy in the absence of drug, suggesting that they do retain certain aspects of an altered phenotype. ZR-75-9a1 cells remain ER and PGR negative and resistant to tamoxifen if cultured in drug-free medium devoid of oestrogenic activity. This finding would suggest that the presence of anti-oestrogenic activity or the absence of oestrogenic activity are equally capable of maintaining the tamoxifen-resistant phenotype of ZR-75-9a1 cells.

Both the R3 (Nawata *et al.*, 1981*b*) and the R27 (Nawata *et al.*, 1981*a*) tamoxifen-resistant variants of the MCF-7 line retain ER although PGR was not inducible in R3 by oestradiol, indicating a defect distal to the ligand binding step. The LY2 MCF-7 variant (Bronzert *et al.*, 1985) expresses a much reduced number of oestrogen binding sites, but retains the ability to respond to oestradiol with growth stimulation. The tamoxifen-resistant variants of MCF-7 may be representative of the clinical situation in which an ER-positive patient fails to respond to tamoxifen. In the case of ZR-75-9a1, a clinical parallel might be the patient who is initially ER-positive and responds, subsequently relapsing with an ER-negative presentation. A number of clinical studies have demonstrated a fall in tumour ER content during endocrine therapy, including tamoxifen treatment (Allegra *et al.*, 1980; Taylor *et al.*, 1982; Nomura *et al.*, 1985). Conversion of an ER-positive tumour to ER negativity as a result of tamoxifen therapy has usually been interpreted as reflecting persistent occupation of ER by the anti-oestrogen. This cannot explain our *in vitro* data since the binding assays were carried out under exchange conditions where ER is detectable in short-term tamoxifen-treated cells and where reversal of tamoxifen's anti-proliferative effects by oestradiol is achieved. Our observation that tamoxifen resistance in ZR-75-9a1 cells can be reversed may also have a clinical parallel among those patients who initially relapse while undergoing anti-oestrogen therapy, but respond to a later challenge with tamoxifen. ZR-75-9a1 cells may be a useful model for furthering our understanding of the development and stability of tamoxifen resistance in human breast cancer.

This work was supported by grants from the Cancer Research Campaign (H.W.v.d.B. and J.M.), Action Cancer (J.N.) and DHSS (N. Ireland) (H.W.v.d.B.).

### References

A'HERN, R.P., WILSON, A.J. & BAUM, M. (1985). Tamoxifen therapy, oestrogen receptor status, and postmenopausal breast cancer. *Lancet*, **i**, 976.

ALLEGRA, J.C., BARLOCK, A., HUFF, K.K. & LIPPMAN, M.E. (1980). Changes in multiple or sequential oestrogen receptor determination. *Cancer*, **45**, 792.

BERTHOIS, Y., KATZENELLENBOGEN, J.A. & KATZENELLENBOGEN, B.S. (1986). Phenol red in tissue culture media is a weak oestrogen: implications concerning the study of oestrogen-responsive cells in culture. *Proc. Natl Acad. Sci. USA*, **83**, 2496.

BRONZERT, D.A., DAVIDSON, N. & LIPPMAN, M. (1986). Estrogen and anti-estrogen resistance in human breast cancer cell lines. *Adv. Exp. Med. Biol.*, **196**, 329.

BRONZERT, D.A., GREENE, D.L. & LIPPMAN, M.E. (1985). Selection and characterisation of a breast cancer cell line resistant to the antiestrogen LY117018. *Endocrinology*, **117**, 1409.

ENGEL, L.W., YOUNG, N.A., TRALKA, T.S., LIPPMAN, M.E., O'BRIEN, S.J. & JOYCE, M.J. (1978). Establishment and characterisation of three new continuous cell lines derived from human breast carcinomas. *Cancer Res.*, **38**, 3352.

GLOVER, J.F., IRWIN, J.T. & DARBRE, P.D. (1988). Interaction of phenol red with oestrogenic and antioestrogenic action on growth of human breast cancer cells ZR-75-1 and T-47-D. *Cancer Res.*, **48**, 3693.

HORWITZ, K.B., MOCKUS, M.B., & LESSEY, B.A. (1982). Variant T47D breast cancer cells with high progesterone receptor levels despite estrogen and antiestrogen resistance. *Cell*, **28**, 633.

LIPTON, A. & MORRIS, I.D. (1986). Calcium antagonism by the antioestrogen tamoxifen. *Cancer Chemother. Pharmacol.*, **18**, 17.

MOURIDSEN, H., PALSHOF, T., PATTERSON, J. & BATTERSBY, L. (1978). Tamoxifen in advanced breast cancer. *Cancer Treat. Rev.*, **5**, 131.

NAWATA, H., BRONZERT, D. & LIPPMAN, M.E. (1981*a*). Isolation and characterisation of a tamoxifen-resistant cell line derived from MCF-7 human breast cancer cells. *J. Biol. Chem.*, **256**, 5016.

NAWATA, H., CHONG, M.T., BRONZERT, D. & LIPPMAN, M.E. (1981*b*). Oestradiol-independent growth of a subline of MCF-7 human breast cancer cells in culture. *J. Biol. Chem.*, **256**, 6895.

NELSON, J., CLARK, R., McFERRAN, N.V. & MURPHY, R.F. (1987). Morpho-functional effects of phenol red on oestrogen-sensitive breast cancer cells. *Biochem. Soc. Trans.*, **15**, 244.

NOMURA, Y., TASHIRO, H. & SHINOZUKA, K. (1985). Changes of steroid hormone receptor content by chemotherapy and/or endocrine therapy in advanced breast cancer. *Cancer*, **55**, 546.

O'BRIAN, C.A., LISKAMP, R.M., SOLOMON, D.H. & WEINSTEIN, B. (1985). Inhibition of protein kinase C by tamoxifen. *Cancer Res.*, **45**, 2462.

PENTZ, S., AMTHOR, S. & VERGANI, G. (1983). Vertical sections of cultivated anchorage-dependent cells for electron microscopy. *J. Microsc.*, **129**, 233.

PENTZ, S., VERGANI, G., AMTHOR, S., HORLER, H. & RICH, I. (1981). A method for electron microscopic preparation of cultured cells (monolayer) in a new test chamber (TSCS-1). *Microsc. Acta*, **84**, 117.

SUTHERLAND, R.L., GREEN, M.D., HALL, R.E., REDDEL, R.R. & TAYLOR, I.W. (1983). Tamoxifen induces accumulation of MCF-7 human mammary carcinoma cells in the G0/G1 phase of the cell cycle. *Eur. J. Cancer Clin. Oncol.*, **19**, 615.

TAYLOR, R.E., POWLES, T.J., HUMPHREYS, J. & 5 others (1982). Effects of endocrine therapy on steroid-receptor content of human breast cancer. *Br. J. Cancer*, **45**, 80.

VAN DEN BERG, H.W., LEAHEY, W.J., LYNCH, M., CLARK, R. & NELSON, J. (1987). Recombinant interferon alpha increases oestrogen receptor expression in human breast cancer cells (ZR-75-1) and sensitises them to the anti-proliferative effects of tamoxifen. *Br. J. Cancer*, **55**, 255.

VIC, P., VIGNON, F., DEROCQ, D. & ROCHEFORT, H. (1982). Effect of oestradiol on the ultrastructure of the MCF-7 human breast cancer cells in culture. *Cancer Res.*, **42**, 667.